# Synaptotagmin 7 Sculpts Short-Term Plasticity at a High Probability Synapse

**DOI:** 10.1523/JNEUROSCI.1756-23.2023

**Published:** 2024-02-28

**Authors:** Delia N. Chiu, Brett C. Carter

**Affiliations:** European Neuroscience Institute Göttingen – A Joint Initiative of the University Medical Center Göttingen and the Max Planck Society, 37077 Göttingen, Germany

## Abstract

Synapses with high release probability (*P_r_*) tend to exhibit short-term synaptic depression. According to the prevailing model, this reflects the temporary depletion of release-ready vesicles after an initial action potential (AP). At the high-*P_r_* layer 4 to layer 2/3 (L4-L2/3) synapse in rodent somatosensory cortex, short-term plasticity appears to contradict the depletion model: depression is absent at interstimulus intervals (ISIs) <50 ms and develops to a maximum at ∼200 ms. To understand the mechanism(s) underlying the biphasic time course of short-term plasticity at this synapse, we used whole-cell electrophysiology and two-photon calcium imaging in acute slices from male and female juvenile mice. We tested several candidate mechanisms including neuromodulation, postsynaptic receptor desensitization, and use-dependent changes in presynaptic AP-evoked calcium. We found that, at single L4-L2/3 synapses, *P_r_* varies as a function of ISI, giving rise to the distinctive short-term plasticity time course. Furthermore, the higher-than-expected *P_r_* at short ISIs depends on expression of synaptotagmin 7 (Syt7). Our results show that two distinct vesicle release processes summate to give rise to short-term plasticity at this synapse: (1) a basal, high-*P_r_* release mechanism that undergoes rapid depression and recovers slowly (τ = ∼3 s) and (2) a Syt7-dependent mechanism that leads to a transient increase in *P_r_* (τ = ∼100 ms) after the initial AP. We thus reveal how these synapses can maintain a very high probability of neurotransmission for multiple APs within a short time frame.

***Key words***: depression; facilitation; short-term plasticity; synaptotagmin 7

## Significance Statement

Release at single L4-L2/3 synapses violates a commonly held synaptic short-term plasticity rule. Although these synapses transmit with very high probability, they do not undergo profound short-term synaptic depression in the tens of milliseconds following an AP. Syt7 is a calcium-sensing protein important for synaptic facilitation and asynchronous release, but not previously known to play a role at high-*P_r_* synapses. We discovered that Syt7-mediated release shapes L4-L2/3 synaptic transmission by effectively counteracting short-term depression for ∼100 ms. We thus establish a molecular basis for a form of information processing at the synaptic level: the combination of these vesicle release properties results in a notch filter, preferentially conveying both very low and very high frequency signals.

## Introduction

Synapses transform presynaptic action potentials (APs) into the release of neurotransmitter that can be detected by postsynaptic cells. Although vesicle release is necessary for signal propagation via chemical synapses, it occurs in a probabilistic fashion (Del Castillo and Katz, 1953; Allen and Stevens, 1994). Synaptic properties such as vesicle release probability (*P_r_*) reflect trade-offs between reliability and flexibility and are tailored to the function of a particular synapse in the context of its circuit (Branco and Staras, 2009; Ribrault et al., 2011). One example of the flexibility built into transmission is synaptic plasticity, where responses to the same presynaptic input change based on prior activity. Plasticity affects synaptic transmission over many time scales ranging from milliseconds to hours or longer (Abbott and Regehr, 2004). A number of pre- and postsynaptic mechanisms of short-term synaptic plasticity have been identified: release can be suppressed or enhanced by neuromodulatory receptor activation (Lovinger et al., 2022); postsynaptic receptors can undergo activity-dependent changes in responsiveness such as desensitization (Trussell and Fischbach, 1989; Chen et al., 2002), facilitation (Rozov and Burnashev, 1999), or saturation (Chen et al., 2002; Foster et al., 2002); and *P_r_* can change due to, for example, alterations in basal (Dittman and Regehr, 1998) or AP-evoked Ca^2+^ (Geiger and Jonas, 2000) or the availability of releasable vesicles (Zucker and Regehr, 2002; Lipstein et al., 2021).

In its simplest form, short-term plasticity can be observed in the responses to just two APs. In this paired-pulse plasticity paradigm, synapses typically show one of two behaviors: facilitation, where the response to the second stimulation is larger than the first, or depression, where the second response is smaller (Zucker and Regehr, 2002). Because facilitation usually occurs at synapses with a relatively low initial *P_r_*, and depression at synapses with a high initial *P_r_*, the paired-pulse ratio (PPR) is often used as a proxy measurement for *P_r_* and consequently presynaptic functional properties (Debanne et al., 1996; Hashimoto and Kano, 1998; Kraushaar and Jonas, 2000; Chen et al., 2004; Bender et al., 2006a; Helmstaedter et al., 2008).

The synapse between layer 4 (L4) and layer 2/3 (L2/3) neurons in rodent somatosensory cortex is a model system to study cortical synaptic processing (Petersen, 2019) as well as the cellular basis of experience-dependent plasticity (Feldman, 2000; Feldman and Brecht, 2005). These synapses are thought to have a uniformly high initial *P_r_* (Castro-Alamancos and Connors, 1997; Feldmeyer et al., 2002; Silver et al., 2003). However, while measuring short-term plasticity at this synapse, we noticed a biphasic time course, where depression developed over ∼200 ms before recovering over several seconds. The lack of depression at the shortest paired-pulse intervals shows that vesicles are available for release immediately after the first stimulation, counter to the vesicle depletion model of paired-pulse depression (PPD). An earlier study of this synapse proposed that neuromodulatory inputs could suppress subsequent synaptic release over a similar time course (Castro-Alamancos and Connors, 1997). If synapse modulation underlies short-term depression at this synapse, this would change our understanding of not only how this synapse processes information but also the causes of short-term synaptic depression. We therefore examined this phenomenon more closely and found that neuromodulation does not account for short-term depression at this synapse. Furthermore, neither postsynaptic receptor responses nor presynaptic AP Ca^2+^ signals displayed short-term plasticity. Functional Ca^2+^ imaging of evoked release at single synapses showed that *P_r_* is initially high at these synapses and follows the same time course as the synaptic responses, depressing at long but not short intervals. This was due to an elevation of release probability at short intervals mediated by synaptotagmin 7 (Syt7). These results show that L4-L2/3 synapses have two separate release mechanisms that shape short-term plasticity: one that drives high-probability vesicle release in response to an initial stimulus, but afterward requires several seconds to recover; and a second, Syt7-dependent mechanism that enables high-probability release for a subsequent stimulus within ∼100 ms.

## Materials and Methods

### Acute slice preparation

Mice of either sex were used in accordance with the Institutional Animal Care and Ethics Committees of the University of Göttingen (T19.3 and T22.20) and with German animal welfare laws. CD1 mice (postnatal day 12–22) were used for all experiments except those in Figure 6. Mice were anesthetized with isoflurane, and the brain was rapidly removed into ice-cold artificial cerebrospinal fluid (ACSF) consisting of the following (in mM): 119 NaCl, 26 NaHCO_3_, 1 NaH_2_PO_4_, 10 glucose, 1.3 Na-ascorbate, 3 Na-pyruvate, 4.2 KCl, 1.2 CaCl_2_, 0.7 MgCl_2_ (Ding et al., 2016), continuously bubbled with 95%/5% O_2_/CO_2_ (chemicals from Carl Roth and Sigma). The brain was then blocked and fixed to a slicing platform with cyanoacrylate glue (Loctite), and slices (270–300 µm) were made using a Leica VT1200S Vibratome. Slices containing somatosensory cortex were incubated at 37°C in ACSF until use.

For experiments testing the function of synaptotagmin-7 (Syt7), we used transgenic mice of either sex that contained a *Syt7* null allele (Maximov et al., 2008) or their wild-type littermates, kindly provided by the Brose lab. Experiments were performed blind to genotype, which was confirmed post hoc by PCR.

### Electrophysiology

Layer 2/3 pyramidal and layer 4 spiny stellate neurons were identified using gradient-contrast video microscopy. Whole-cell recordings were obtained using borosilicate glass patch pipettes (open-tip resistance, 2–6 MΩ). For voltage-clamp recordings, the internal solution consisted of the following (in mM): 130 Cs-methanesulfonate, 10 NaCl, 10 HEPES, 4 MgCl_2_, 14 Na-phosphocreatine, 4 ATP, 0.4 GTP, 0.1 EGTA, pH adjusted to 7.3 with CsOH. Internal solution used for current-clamp recordings consisted of the following (in mM): 128 K-gluconate, 10 NaCl, 10 HEPES, 4 MgCl_2_, 14 Na-phosphocreatine, 4 ATP, 0.4 GTP, 0.5 Fluo-4FF (Bio-Techne), and 0.015 Alexa Fluor 594 (Bio-Techne) for axon imaging experiments (Fig. 4) or 0.3 Fluo-5F (Bio-Techne) and 0.01 Alexa Fluor 594 for synapse imaging experiments (Fig. 5), pH adjusted to 7.3 with KOH. For voltage-clamp imaging experiments, internal solution consisted of the following (in mM): 135 Cs-gluconate, 10 HEPES, 4 MgCl_2_, 10 Na-phosphocreatine, 4 ATP, 0.4 GTP, 0.3 Fluo-5F, and 0.01 Alexa Fluor 594.

Electrophysiology recordings were filtered at 10 kHz and collected at 50 kHz using a MultiClamp 700B amplifier. Reported membrane potentials have been corrected for the junction potential (−8 mV relative to ACSF for Cs-based internal solutions and −11 mV for K-based internals). All experiments were performed in continuously bubbled ACSF (2 ml/min) at 32–35°C.

For PPR measurements, the response to a single stimulus was measured, followed by trials with each interstimulus interval (ISI) from shortest to longest. The time between each trial was 10 s, and the entire series was repeated 5–10 times for a given condition. Synaptic currents were averaged and measured over 0.5 ms at the current peak. The response to the second stimulation for short ISIs was isolated by subtracting the response to a single stimulation before measurement.

### Pharmacology

Isolation of α-amino-3-hydroxy-5-methyl-4-isoxazolepropionic acid receptor (AMPAR) currents (Fig. 3) was accomplished using 50 µM D-AP5 (from 50 mM stock solution in water; Hello Bio) to inhibit *N*-methyl-D-aspartic acid receptors (NMDARs) and 50 µM picrotoxin (from 500 mM stock solution in DMSO; Abcam) to inhibit GABA_A_Rs while holding the neuron near its resting potential, at −78 mV. Isolation of NMDAR currents (Fig. 3) was accomplished by inhibiting AMPARs with 5 µM NBQX (from 50 mM stock solution in water; Hello Bio) and 50 µM picrotoxin while holding at +32 mV to relieve Mg^2+^ block of the NMDAR ion channel.

To test for neuromodulator effects on short-term depression (Fig. 2), antagonists were added to the bath and were continuously present during the experiments. To block metabotropic glutamate receptor (mGluR) activity, (*S*)-α-Methyl-4-carboxyphenylglycine (MCPG, dissolved in ACSF; Hello Bio) was used at a concentration of 0.5 or 1 mM. To block GABA_B_Rs, CGP-55845 (50 mM stock solution in DMSO; Tocris) was used at a concentration of 10 µM. To block CB_1_Rs, AM-251 (10 mM stock solution in DMSO; Hello Bio) was used at 1 µM. To block A_1_Rs, DPCPX (5 mM stock solution in DMSO; Hello Bio) was used at 1 µM.

For glutamate uncaging experiments, 2.5 mM MNI-glutamate (Tocris) was dissolved in ACSF and was continuously present in the bath solution.

### Two-photon laser scanning microscopy

Imaging was performed using a Bruker Ultima In Vitro BX51 system (Bruker). The imaging laser (Coherent Ultra II) was tuned to 810 or 840 nm. A second laser, tuned to 720 nm, was used for focal uncaging of MNI-glutamate. Fluorescence emission was separated into red (epifluorescence) and green (epi- and transfluorescence) channels and detected with GaAsP photomultiplier tubes (H7422PA, Hamamatsu).

To allow for diffusion of the fluorescent dyes into the cell, imaging began no sooner than 20 min after break-in. Alexa Fluor 594, the red fluorescent dye, was used to measure neuronal morphology and locate structures of interest. The green fluorescence channel was used to monitor Ca^2+^-sensitive fluorescence (Fluo-5F or Fluo-4FF). For synapse imaging, the dendritic arbor of a L2/3 neuron was systematically inspected while stimulating in L4 until a response was seen in the green, Ca^2+^-sensitive fluorescence signal. Once a responsive synapse was found, the fluorescent signal as a function of time was measured by repetitive scans across the structure at 0.5–2.0 kHz. Fluorescence signals were quantified as the change in Ca^2+^-sensitive green fluorescence over time, *G(t)*, relative to its baseline measured over ∼20 ms prior to stimulation, *G(0)*, normalized to the red fluorescence signal, *R*:

$$\frac{\Delta G}{R}=\;\frac{G(t)-G(0)}{R},$$

Δ*G*/*R* for the first pulse was calculated as the average of 2–4 ms at the peak of the fluorescence transient. For the second pulse, average ΔG/R traces were baselined to 3–5 ms before stimulation before measurement.

### Experimental design and statistical analysis

**Figure 1.**
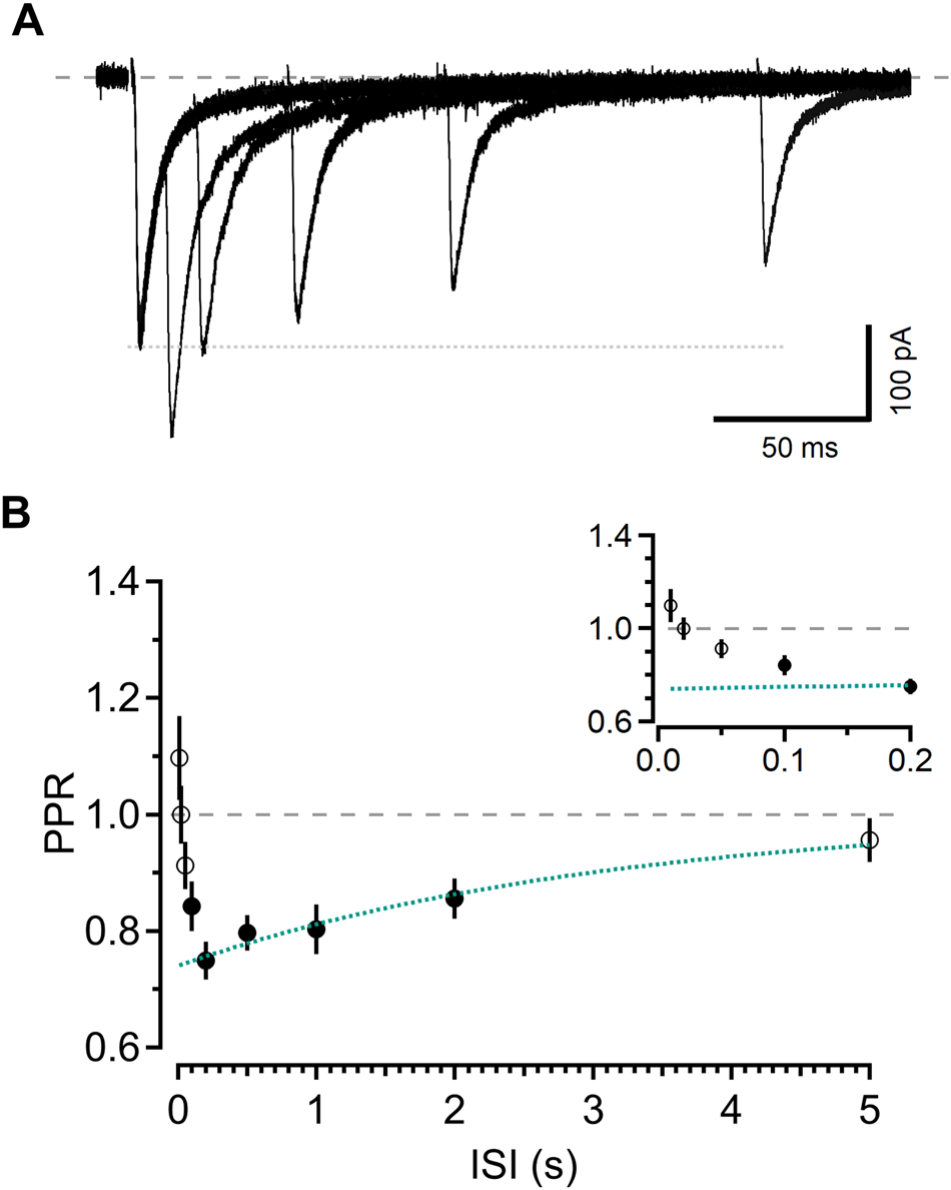
Short-term synaptic plasticity at barrel cortex L4-L2/3 synapses. ***A***, L2/3 EPSCs recorded at −78 mV elicited by pairs of stimuli (black) delivered to L4 at various ISIs. Each trace is the average of ten sweeps. Dotted line indicates amplitude of single stimulation response. ***B***, Summary of PPR as a function of ISI (*n* = 19). Green dotted line is exponential fit from 200 ms to 5 s (τ = 3.12 s). Symbols here and in all subsequent figures represent mean ± SEM. Closed symbols indicate ISIs at which EPSCs were depressed relative to first response. PPR was analyzed using repeated-measures ANOVA (*F*_(9,162)_ = 11.454; *p* < 0.0001) followed by Dunnett’s test, *p* < 0.05 for 2 s, *p* < 0.01 for 100 ms, 200 ms, 500 ms, 1 s. Inset shows the same data on an expanded timescale.

**Figure 2.**
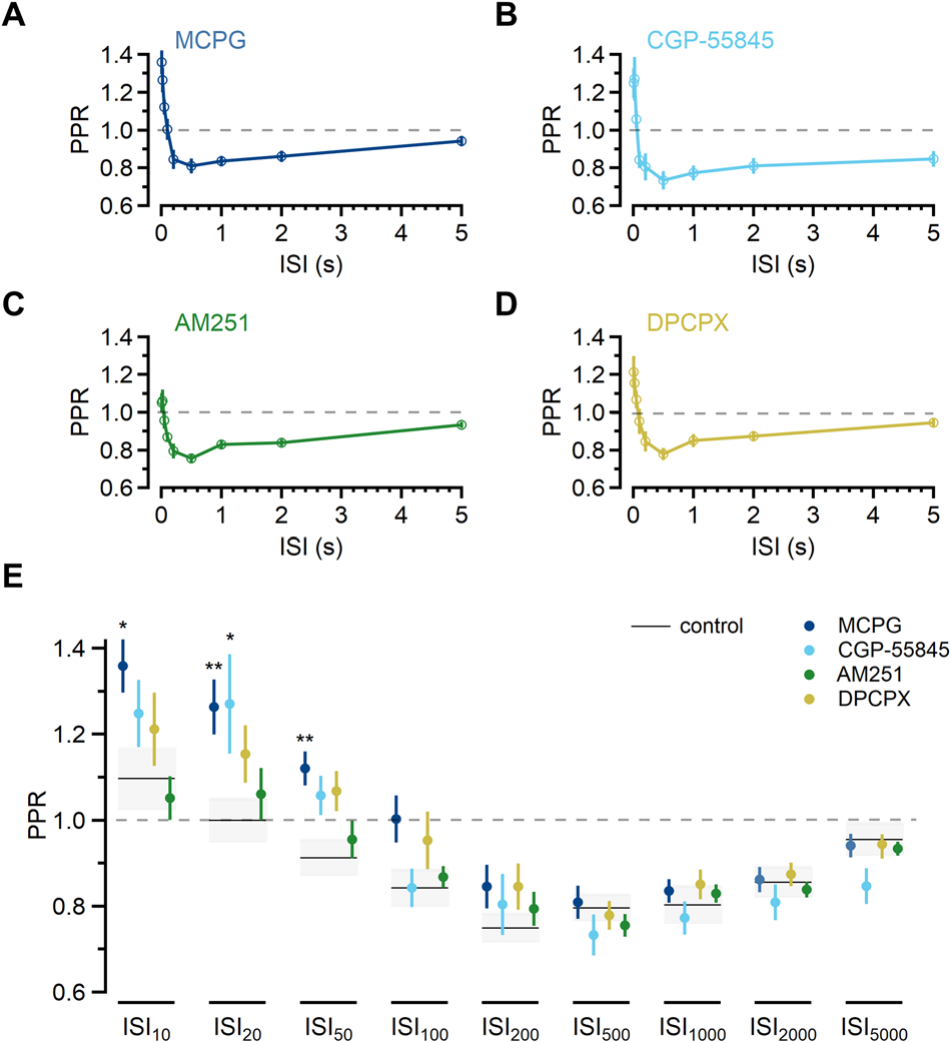
Synaptic depression is not mediated by common neuromodulatory GPCRs. ***A–D***, PPR was largely unchanged by the presence of ***A***, the nonselective mGluR antagonist MCPG (blue; 0.5 mM; *n* = 19); ***B***, the GABA_B_R antagonist CGP-55845 (cyan; 1 µM; *n* = 9); ***C***, the CB_1_R antagonist AM251 (green; 1 µM; *n* = 19); or ***D***, the A_1_R antagonist DPCPX (yellow; 1 µM; *n* = 14). Symbols denote mean ± SEM. ***E***, Summary of PPR in the different conditions for each ISI. Black lines and gray bands indicate mean PPR ± SEM in control ACSF (data from Fig. 1). Symbols denote mean ± SEM. The effect of antagonists on PPR was analyzed using one-way ANOVA for each ISI, followed by Dunnett’s test for ISI_10_, ISI_20_, and ISI_50_, *p ≤ 0.05, **p ≤ 0.01.

**Figure 3.**
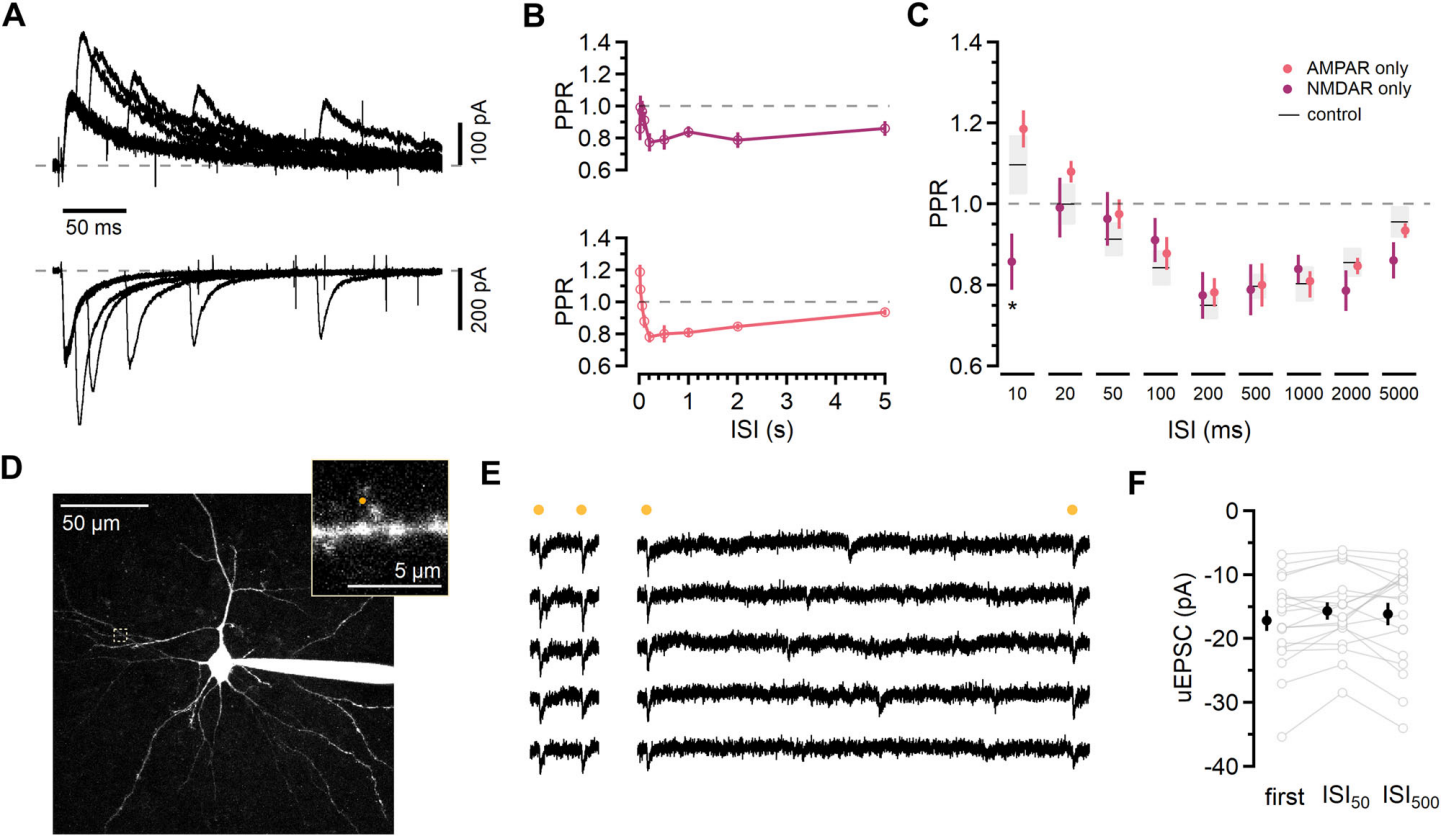
Postsynaptic L2/3 receptor properties do not explain short-term plasticity time course. ***A***, Pairs of evoked NMDAR EPSCs (top traces) measured at +32 mV in the presence of NBQX (10 µM) and picrotoxin (50 µM). AMPAR currents (bottom traces) elicited by paired-pulse stimulation measured at −78 mV in the presence of D-AP5 (50 µM) and picrotoxin (50 µM). ***B***, PPR of NMDAR-mediated responses (top; purple) and PPR of AMPAR-mediated responses (bottom; red) at the ISIs indicated. Symbols denote mean ± SEM. ***C***, Summary graph of PPR of NMDAR and AMPAR EPSCs. Symbols denote mean ± SEM. Black lines and gray bands indicate mean PPR ± SEM in control ACSF. At ISI_10_ PPR of NMDAR-mediated responses was less than control PPR. AMPAR-only PPR and NMDAR-only PPR were compared with control PPR for each ISI using one-way ANOVA followed by Dunnett’s test for ISI_10_, *p ≤ 0.05. ***D***, Two-photon maximum projection image of a L2/3 neuron and dendritic segment (inset). The uncaging laser was directed to the spot indicated in orange. ***E***, Glutamate uncaging responses (uEPSCs) from the spine shown in ***C*** with an ISI of 50 ms (left) and 500 ms (right). Orange symbols indicate the timing of uncaging pulses (1 ms). ***F***, Comparison of the initial uEPSC with uEPSCs at ISI_50_ and ISI_500_. Peak uEPSC amplitudes from individual spines are shown with open symbols (gray), mean ± SEM with closed symbols (black). Amplitudes were analyzed using repeated-measures ANOVA (*F*_(2,36)_ = 1.929; *p* = 0.16).

**Figure 4.**
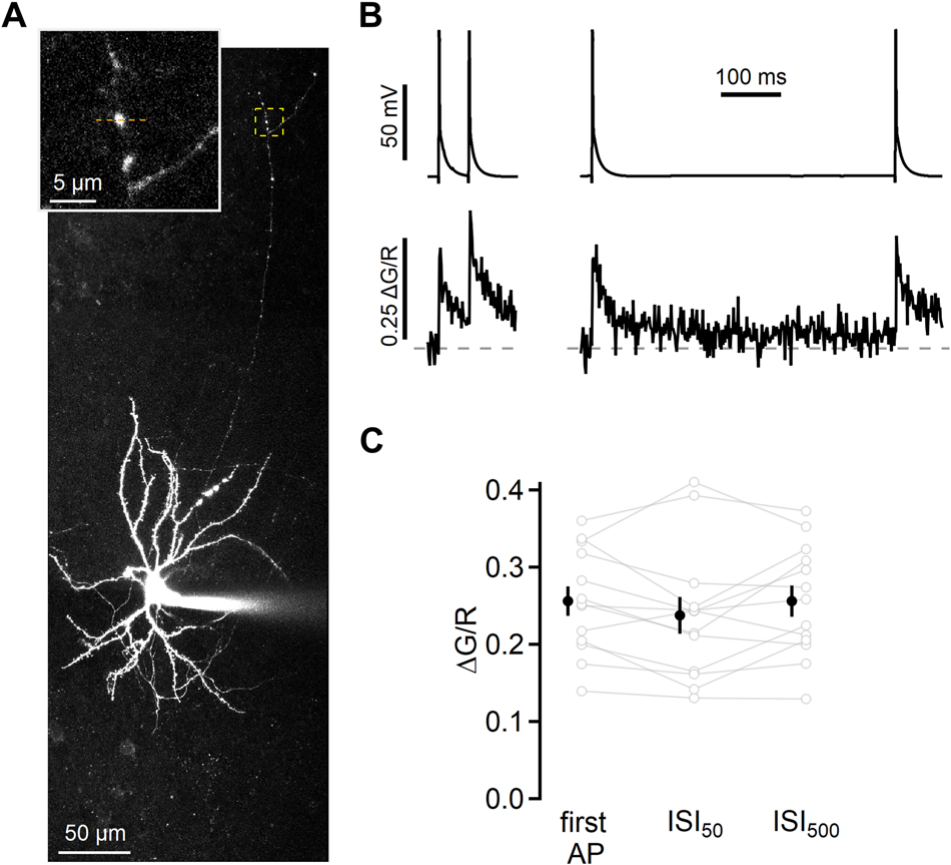
Presynaptic L4 AP Ca^2+^ influx is similar at different ISIs. ***A***, Two-photon maximum projection image of L4 cell and distal axon varicosity (boxed region and inset) in which AP Ca^2+^ signals were measured. ***B***, APs generated by current injection in the L4 cell (top) and measurements of the change in Fluo-4FF signal relative to the Alexa Fluor 594 signal (ΔG/R; bottom) from line scans across the varicosity indicated by the orange dashed line in ***A***. Traces are the mean of 69 and 71 repetitions at 50 ms and 500 ms intervals, respectively. ***C***, Comparison of the AP-evoked Ca^2+^ transients for the first AP, at ISI_50_, and at ISI_500_. Data from individual boutons are shown with open symbols (gray), the mean ± SEM with closed symbols (black). Transients were analyzed using repeated-measures ANOVA (*F*_(2,24)_ = 1.871; *p* = 0.18).

**Figure 5.**
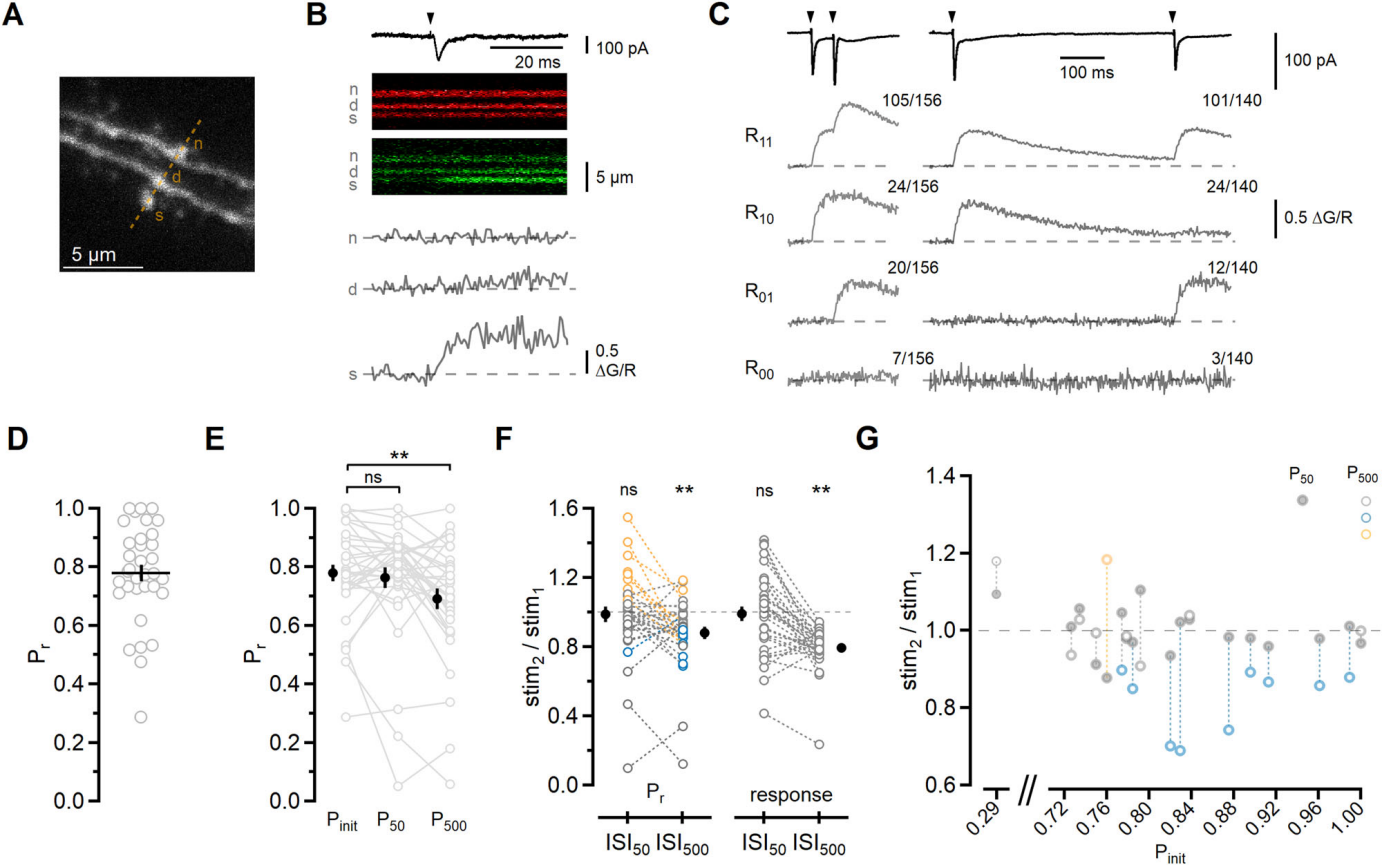
Optical detection of synaptic release shows *P_r_* depends on ISI for pairs of stimuli. ***A***, Maximum projection image of L2/3 basal dendrites with spine (s) that responded to extracellular L4 stimulation. ***B***, In the example shown, stimulation (arrowhead) elicited an EPSC (top trace). Below is the fluorescence in red and green channels collected using repeated scans across the area indicated by the orange dashed line in ***A***. The change in green over red fluorescence (ΔG/R; gray traces) shows a large, rapid increase in Fluo-5F fluorescence in the responding spine (s). A much smaller and slower increase can be seen in the parent dendrite (d), and no relative change in fluorescence is observed in a spine (n) on a neighboring dendritic branch. ***C***, Average evoked EPSCs from the cell in ***A*** for ISI_50_ and ISI_500_ (156 and 140 trials, respectively). Below each average EPSC trace are average Δ*G*/*R* traces showing the four possible response types (from top to bottom: *R*_11_, *R*_01_, *R*_10_, and *R*_00_) and the fraction of trials which each average represents. ***D***, Summary of *P*_init_ for each synapse (gray symbols), calculated by dividing the number trials responding to the initial stimulation by the total number of trials. Black line indicates mean ± SEM. ***E***, Comparison of *P*_init_, *P*_50_, and *P*_500_. Data from individual experiments are shown with open symbols (gray) and the mean ± SEM with closed symbols (black). Statistical analysis was done using repeated-measures ANOVA (*F*_(2,66)_ = 7.821; *p* = 0.0009) followed by Dunnett’s test; ns, not significant; **p ≤ 0.01. ***F***, Left, Summary of *P*_50_ and *P*_500_ normalized to *P*_init_. Colors indicate whether *P*_50_ and *P*_500_ were within the 95% CI of *P*_init_ for that synapse (gray); higher than the 95% CI (orange); or below the 95% CI (blue). Right, summary of normalized electrophysiology responses from these experiments. Open symbols are individual experiments and closed symbols are mean ± SEM. Statistical analysis was done using repeated-measures ANOVA (*P_r_*: *F*_(2,66)_ = 5.809, *p* = 0.005; response: *F*_(2,66)_ = 29.589, *p* < 0.0001) followed by Dunnett’s test; ns, not significant; **p ≤ 0.01. ***G***, Summary of relative changes in *P_r_* versus *P*_init_ for the 18 synapses in which *P*_50_ (closed symbols) was within the 95% CI of *P*_init_. *P*_50_ and *P*_500_ (open symbols) are normalized to *P*_init_, as in ***F***. Colors as in ***F***.

**Figure 6.**
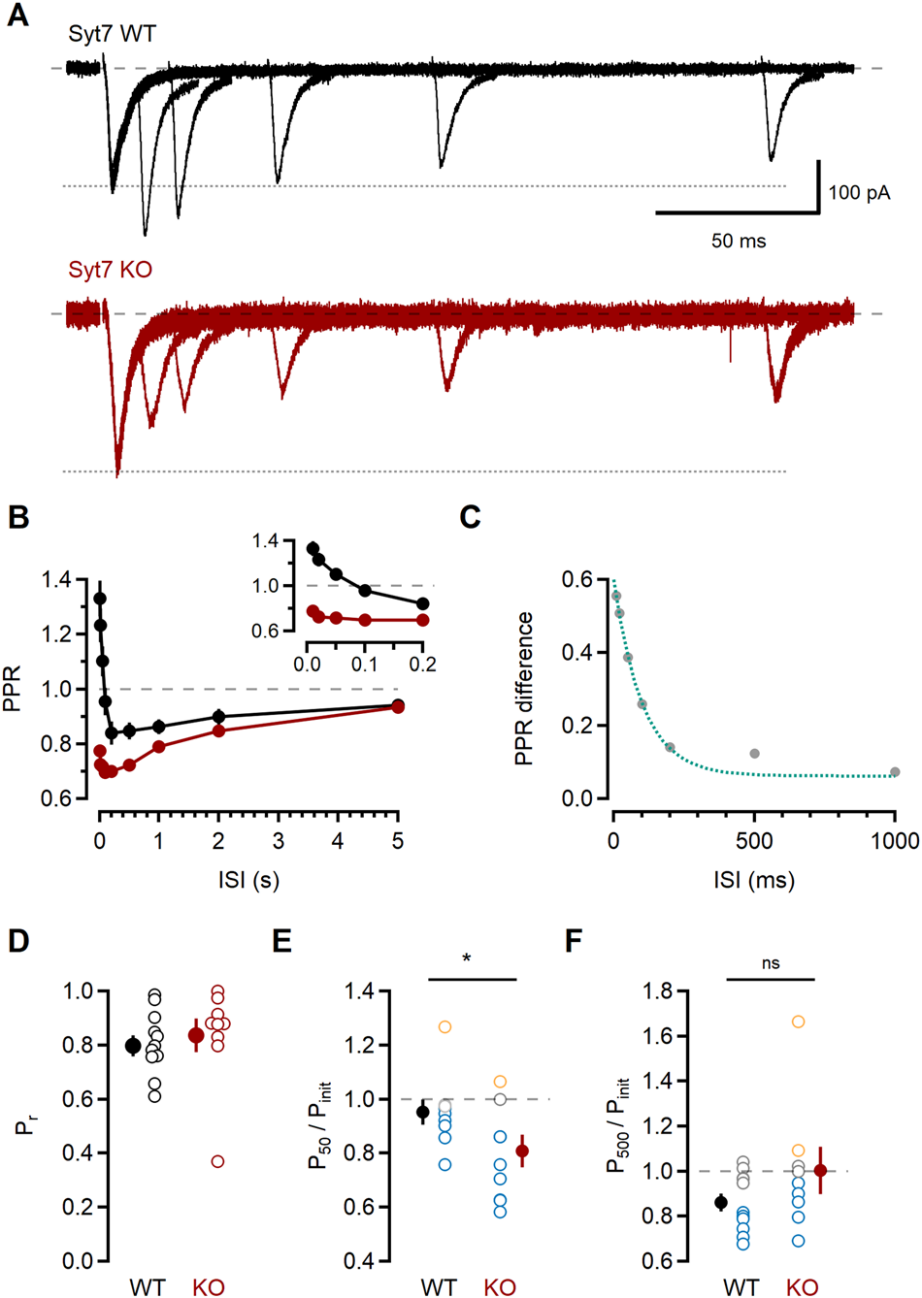
Synaptotagmin 7 counteracts PPD at short ISIs. ***A***, L2/3 responses to pairs of stimuli delivered at various intervals in recordings from Syt7-KO mice (bottom traces, red) and WT littermates (top traces, black). Each trace is the average of ten trials. Dotted lines correspond to amplitude of single stimulation response. ***B***, PPR as a function of ISI. PPR in recordings from Syt7-KO mice (red) is reduced compared with WT littermates (black). Inset, expanded timescale shows PPD at brief ISIs in Syt7-KO but not Syt7-WT responses. ***C***, The difference in PPR between Syt7-KO and WT recordings for each ISI was plotted (gray) and fitted with a single exponential (green dotted line; τ = 101.4 ms). ***D***, Summary of *P*_init_ calculated in imaging experiments for Syt7-KO (red symbols) and Syt7-WT synapses (black symbols). Statistical analysis was done using paired *t* test; *p ≤ 0.05. ***E***,***F***, Summary of *P*_50_ (***E***) and *P*_500_ (***F***) normalized to *P*_init_ for Syt7-WT and Syt7-KO synapses. Open symbols are individual experiments, and colors indicate whether *P*_50_ and *P*_500_ were within the 95% CI of *P*_init_ for that synapse (gray); higher than the 95% CI (orange); or below the 95% CI (blue). Closed symbols are mean ± SEM for Syt7-WT (black) and Syt7-KO (red) experiments. Statistical analysis was done using paired *t* test; ns, not significant.

Values are presented as mean ± standard error of the mean (SEM). To determine whether paired-pulse plasticity was present at the ISIs tested (Fig. 1), we used a one-way repeated-measures ANOVA to compare PPR for each ISI to the normalized single pulse response. This was followed by Dunnett’s test to determine at which ISI PPR was different from the initial pulse. Similarly, to determine at which ISIs the second response differed from the response to a single stimulation (Figs. 3*F*, 4*C*, 5*E*, 6*E*), we used the repeated-measures ANOVA test. To determine whether a pharmacological manipulation changed PPR relative to control (Figs. 2*E*, 3*C*), we compared all groups for each ISI separately using a one-way ANOVA test. In all cases where the ANOVA *p* value was <0.05, we used Dunnett’s test to compare the relevant groups with the control group (values for the first stimulation or values for control ACSF, as appropriate); otherwise, no further tests were performed. To compare PPR between Syt7-WT and Syt7-KO animals (Fig. 6*B*), we used an unpaired *t* test for each ISI, followed by the Holm–Sidak test to correct for multiple comparisons; the reported *p* values are uncorrected. For comparisons between Syt7-WT and Syt7-KO (Fig. 6*D*,*E*), we used unpaired *t* tests. Threshold for significance was set at 0.05. In figure legends, statistical notation is as follows: ns, *p* > 0.05; *p ≤ 0.05; **p ≤ 0.01.

Probability of release was estimated by dividing the number of trials with a success on the first stimulation by the total number of trials. The 95% confidence intervals for this measurement were calculated using the inverse binomial distribution function.

Data collection and analysis were not performed blind to the conditions of the experiments, except for experiments using Syt7-KO and -WT mice (Fig. 6). Randomization was not used. No statistical methods were used to predetermine sample sizes, but our sample sizes are similar to those generally employed in the field.

### Resource availability

Requests for further information, resources, or data should be directed to and will be fulfilled by the lead contact, Brett Carter (b.carter@eni-g.de).

## Results

### L4-L2/3 responses depress at longer but not shorter interstimulus intervals

To examine short-term plasticity at glutamatergic L4-L2/3 synapses, we made whole-cell patch-clamp recordings from visually identified L2/3 neurons in acute slices of mouse barrel cortex using physiological ionic conditions (Ding et al., 2016). A bipolar theta-glass stimulation electrode was placed in L4 of the same cortical column to evoke excitatory postsynaptic currents (EPSCs; Fig. 1*A*) that arise through activation of AMPARs and NMDARs (Feldman, 2000; Feldmeyer et al., 2002; Chiu and Carter, 2022).

Short-term synaptic plasticity was investigated using pairs of stimuli at a variety of ISIs and quantified as the ratio of the peak amplitude of the second EPSC relative to the first (PPR). We analyzed the responses using a one-way repeated-measures ANOVA to identify which ISIs were associated with paired-pulse plasticity (*F*_(9,162)_ = 11.454; *p* < 0.0001). On average, the response to the second stimulation was not different from the first at ISIs shorter than 100 ms (Fig. 1*B*). However, at most longer intervals tested, there was significant depression (Fig. 1*B*; PPR for ISI*_t_*, where *t* is the interval, in ms, between stimuli, ISI_10_: 1.10 ± 0.07, *p* > 0.10; ISI_20_: 1.00 ± 0.05, *p* > 0.10; ISI_50_: 0.91 ± 0.04, *p* > 0.10; ISI_100_: 0.84 ± 0.04, *p* < 0.01; ISI_200_: 0.75 ± 0.03, *p* < 0.01; ISI_500_: 0.80 ± 0.03, *p* < 0.01; ISI_1000_: 0.80 ± 0.04, *p* < 0.01; ISI_2000_: 0.86 ± 0.03, *p* > 0.05; ISI_5000_: 0.96 ± 0.04, *p* > 0.10; *n* = 19; Dunnett’s test). The recovery to baseline from 200 ms to 5 s was well fitted by a single exponential function with a time constant of 3.1 s (Fig. 1*B*), but there was a large deviation from this fitted relationship at intervals shorter than ∼100 ms.

Typically, synapses with an initially high *P_r_* show PPD that is maximal immediately after the first stimulus before recovering over seconds (Dittman and Regehr, 1998; Hashimoto and Kano, 1998; Silver et al., 1998; Murphy et al., 2004). The canonical explanation for this type of plasticity is that, with high initial *P_r_*, releasable vesicles are depleted by the first stimulus, and the recovery time course corresponds to vesicle replenishment (Curtis and Eccles, 1960; Zucker and Regehr, 2002; Foster and Regehr, 2004; Regehr, 2012). The lack of PPD at the briefest intervals was therefore puzzling, as L4-L2/3 synapses have a very high initial *P_r_* (Castro-Alamancos and Connors, 1997; Feldmeyer et al., 2002; Silver et al., 2003).

### Short-term depression is not caused by neuromodulation

The non-monotonic time course of short-term plasticity we observed has been previously observed in L2/3 field EPSP recordings (Castro-Alamancos and Connors, 1997) and paired recordings between individual L4 and L2/3 neurons (Feldmeyer et al., 2002). Noting the unexpected time course of PPD, Castro-Alamancos and Connors (1997) speculated that the latency could be evidence of neuromodulatory inputs that, activated by the first stimulation, suppress subsequent synaptic release. G-protein-coupled receptors (GPCRs) are widely expressed, and act homo- or heterosynaptically with a latency of tens to hundreds of millisecond (Lovinger et al., 2022). To determine whether short-term depression of L4-L2/3 responses is caused by neuromodulator activity, we turned our attention to GPCRs that could plausibly be activated by the first stimulation to inhibit synaptic release.

Glutamate itself can exert an inhibitory effect on subsequent release via activation of presynaptic metabotropic glutamate receptors (mGluRs; Burke and Hablitz, 1994), and a single AP leads to glutamate release with high probability at L4-L2/3 synapses (Castro-Alamancos and Connors, 1997; Feldmeyer et al., 2002; Silver et al., 2003). To test whether mGluR signaling accounts for L4-L2/3 short-term depression, we recorded pairs of EPSCs in the presence of the nonselective mGluR antagonist MCPG (0.5 or 1 mM; Fig. 2*A*; *n* = 19) and found PPD to be unaffected.

A second candidate neuromodulator is γ-aminobutyric acid (GABA) acting through metabotropic GABA_B_ receptors (GABA_B_Rs). GABA_B_Rs inhibit release at a number of presynaptic terminals in the brain (Bowery et al., 2002) and function over a similar time course as the short-term plasticity seen here (Davies et al., 1990). GABAergic interneurons are important components of cortical microcircuits, providing inhibitory control over neuronal integration and output throughout the cortex (Tremblay et al., 2016), including the somatosensory cortex (Staiger and Petersen, 2021). In our preparation, GABAergic interneurons could be activated either directly by stimulation or indirectly through a disynaptic connection (Helmstaedter et al., 2008; Meyer et al., 2011); however, PPD measured with the GABA_B_R antagonist CGP-55845 (10 µM) present was unaffected (Fig. 2*B*; *n* = 9).

Endocannabinoid signaling is known to suppress synaptic responses through the action of the CB1 receptor (CB1R), including at cortical synapses (Auclair et al., 2000; Schlicker and Kathmann, 2001; Domenici et al., 2006; Li et al., 2010), and L2/3 pyramidal neurons can generate endocannabinoids in response to activity (Bender et al., 2006b; Nevian and Sakmann, 2006; Min and Nevian, 2012). To test whether endocannabinoid signaling through CB1R accounts for PPD, we recorded responses in the presence of the CB1R antagonist/inverse agonist AM251 and found no effect (1 µM; Fig. 2*C*; *n* = 19).

Another candidate neuromodulator is adenosine, which, acting through A_1_ receptors, inhibits synaptic release (Ribeiro, 1995; Lovatt et al., 2012; Qi et al., 2017). Stimulation in L4 could lead to adenosine release from neurons or astrocytes (Lovatt et al., 2012), and adenosine receptor activity has been shown to inhibit transmission at barrel cortex L4-L2/3 synapses in an activity-dependent manner (Martínez-Gallego et al., 2022). In our experiments, the A_1_ receptor antagonist DPCPX (1 µM) did not affect the time course of short-term plasticity (Fig. 2*D*; *n* = 14).

To analyze whether these pharmacological manipulations affected PPR relative to control, we used one-way ANOVA to compare all conditions for each ISI (Fig. 2*E*; ISI_10_ *F*_(4,75)_ = 3.611, *p* = 0.01; ISI_20_ *F*_(4,75)_ = 3.331, *p* = 0.01; ISI_50_ *F*_(4,75)_ = 4.316, *p* = 0.003; all other ISIs *p* > 0.05). The results of these tests indicated that there was no difference among the groups for ISIs ≥ 100 ms. For ISI_10_, ISI_20_, and ISI_50_, we used Dunnett’s test to determine which antagonists changed PPR relative to control (Fig. 1) and found that PPR was increased in MCPG at each (ISI_10_: 1.36 ± 0.06, *p* = 0.02; ISI_20_: 1.26 ± 0.06, *p* = 0.01; ISI_50_: 1.12 ± 0.04, *p* < 0.01, *n* = 19; Dunnett’s test). PPR also increased for ISI_20_ in the presence of CGP-55845 (1.27 ± 0.12; *p* = 0.05; *n* = 9; Dunnett’s test), consistent with GABA_B_R-mediated suppression of release (Davies et al., 1990). No other difference in paired-pulse plasticity was detected at any ISI. These results indicate that mGluR and GABA_B_R signaling may be constitutively active or are recruited with high-frequency stimulation (Helmstaedter et al., 2008; Viaene et al., 2013). However, because the PPD observed in control ACSF at ISI_100_, ISI_200_, ISI_500_, and ISI_1000_ was unaffected by inhibition of mGluRs, GABA_B_Rs, A_1_Rs, or CB_1_Rs, we concluded that none of these neuromodulatory pathways underlies short-term depression at this synapse.

While other neuromodulatory systems are present in cortical synapses (Radnikow and Feldmeyer, 2018), the fact that this short-term plasticity time course is observed in the reduced acute slice preparation and can also be seen in paired recordings from L4-L2/3 neurons (Feldmeyer et al., 2002) limits the possible modulatory signals that could account for these dynamics and indicates that its determinants are likely intrinsic to the L4-L2/3 synapse itself.

### Short-term plasticity does not arise from postsynaptic receptor properties

Ionotropic glutamate receptors have dynamic properties that can influence short-term plasticity (Chen et al., 2002). Both AMPARs and NMDARs undergo desensitization (Mayer et al., 1984; Trussell and Fischbach, 1989; Mennerick and Zorumski, 1996; Rozov et al., 2001) and receptor saturation can lead to diminished NMDAR responses to successive stimuli (Chen et al., 2002). Alternatively, AMPAR facilitation at brief ISIs (Rozov and Burnashev, 1999) could mask short-term depression, giving rise to the seeming delay in PPD onset.

To test whether postsynaptic receptor dynamics underlie short-term plasticity at L4-L2/3 synapses, we measured PPR in conditions to isolate either NMDARs or AMPARs (Fig. 3*A*). NMDAR responses were isolated by blocking AMPARs with NBQX (10 µM) and GABA_A_Rs with picrotoxin (50 µM) while holding the L2/3 neuron at +32 mV to relieve Mg^2+^ block (Mayer et al., 1984). AMPAR responses were isolated by blocking NMDARs with D-AP5 (50 µM) while holding the L2/3 neuron at −78 mV, likewise in the presence of 50 µM picrotoxin. The PPR time course was similar in both conditions (Fig. 3*B,C*). Compared with PPR in control ACSF (Fig. 1), there was no difference with either receptor type blocked except for at ISI_10_ (*F*_(2,34)_ = 4.121; *p* = 0.02; one-way ANOVA), at which PPR for NMDAR-mediated currents was decreased (0.85 ± 0.07; *n* = 10; *p* = 0.047; Dunnett’s test).

NMDAR and AMPAR processes have distinct time courses, yet isolating either receptor type did not alter the pattern of short-term plasticity. These results are consistent with a presynaptic locus of short-term plasticity (Chen et al., 2002; Murphy et al., 2004). However, these postsynaptic measurements rely on stimulated presynaptic release, which can confound their interpretation. To test more directly whether postsynaptic receptor properties underlie the pattern of short-term plasticity at L4-L2/3, we used two-photon photolysis of caged glutamate to bypass the contribution of presynaptic release mechanisms while mimicking the spatial and temporal aspects of vesicular glutamate release (Carter and Sabatini, 2004; Fig. 3*D,E*).

Recordings were made from L2/3 neurons loaded with the red fluorophore Alexa Fluor 594 (10 µM) through the patch pipette in the presence of 2.5 mM MNI-glutamate (Fig. 3*D*). We selected spines along basal dendrites (mean geometric distance from soma 65.5 ± 7.1 µm; *n* = 19), where L4-L2/3 synapses are known to occur (Feldmeyer et al., 2002). For each dendritic spine, uncaging laser power was adjusted such that a brief (1 ms) pulse elicited a rapid inward current (uEPSC; Fig. 3*E,F*). We tested the responses to pairs of uncaging pulses using ISI_50_, an interval at which no PPD is observed, and ISI_500_, an interval at which there is significant depression in evoked responses (Fig. 1). In contrast to synaptic EPSCs, the amplitude of second uEPSC was not different from the first for either ISI (Fig. 3*F*; first uEPSC: −17.2 ± 1.6 pA; ISI_50_: −15.7 ± 1.4 pA; ISI_500_: −16.2 pA ± 1.8 pA; *n* = 19; *F*_(2,36)_ = 1.929; *p* = 0.16; repeated-measures ANOVA).

Together, these results indicate that the short-term plasticity time course observed with synaptic stimulation does not arise from postsynaptic receptor desensitization, saturation, or facilitation. Having ruled out likely neuromodulatory and postsynaptic mechanisms and that could underlie the short-term plasticity time course, we next examined intrinsic presynaptic processes governing synaptic vesicle release.

### L4 axonal Ca2+ transient amplitude is unchanged for pairs of APs

One possible presynaptic source of differential release to paired stimuli is alteration of AP-evoked Ca^2+^ influx. Evoked vesicle release depends on Ca^2+^ influx through voltage-dependent Ca^2+^ channels (VDCCs) into the presynaptic terminal (Katz and Miledi, 1967). VDCCs can undergo both activity-dependent facilitation and inactivation (Catterall and Few, 2008), leading to changes in evoked release (Xu and Wu, 2005; Mochida et al., 2008). In addition, prior activity can change AP shape (Geiger and Jonas, 2000), potentially altering AP-evoked Ca^2+^ influx (Sabatini and Regehr, 1996) on an AP-to-AP basis. If Ca^2+^ entry is enhanced for APs at short intervals or reduced at long intervals, this might explain the difference in postsynaptic response sizes.

To monitor AP-induced Ca^2+^ influx, whole-cell current-clamp recordings were made from L4 spiny stellate neurons filled with the low-affinity Ca^2+^-sensitive dye Fluo-4FF (500 µM), along with Alexa Fluor 594 (15 µM) to image neuronal morphology. Axons were identified by their thin morphology and the presence of varicosities, which are presumptive synaptic boutons (Fig. 4*A*). Line scan measurements across varicosities from axon collaterals that traversed L2/3 toward the pial surface (average geometric distance from the L4 soma: 212.8 ± 29.7 µm; *n* = 13 varicosities from eight cells) revealed fast Ca^2+^ transients when APs were elicited with brief current injections (1–1.5 ms), consistent with AP-evoked VDCC activation (Koester and Sakmann, 2000; Fig. 4*B*). Ca^2+^ transients were quantified as the change in green relative to red fluorescence (Δ*G*/*R*). In pairs of APs elicited at either ISI_50_ or ISI_500_, the Ca^2+^ signal was the same for the first and second AP (Fig. 4*C*; first AP: 0.26 ± 0.02; ISI_50_: 0.24 ± 0.02; ISI_500_: 0.26 ± 0.02; *n* = 13; *F*_(2,24)_ = 1.871; *p* = 0.18; repeated-measures ANOVA). These results indicate that short-term synaptic plasticity at these synapses does not arise from modulation of AP-evoked Ca^2+^ in L4 axons.

### Release probability depends on ISI at single synapses

The results of the preceding experiments point to a short-term plasticity mechanism that is both presynaptic and downstream of AP-evoked Ca^2+^ influx. Although L4-L2/3 synapses have a high initial *P_r_* (Castro-Alamancos and Connors, 1997; Feldmeyer et al., 2002; Silver et al., 2003) that would preclude facilitation, and the lack of depression at short intervals appears to contradict the canonical explanation of short-term depression by depletion of available release-competent vesicles, overlapping processes of facilitation and depression could combine to generate the non-monotonic short-term plasticity time course in at least two (non-mutually exclusive) ways. The synapses activated by our stimulation could be heterogeneous, in that some have a high initial release probability and depress, while others have a low initial release probability and facilitate; simultaneous activation of these separate synapses could summate into the whole-cell response (Castro-Alamancos and Connors, 1997). A second possibility is that both processes occur in the same synapse (Regehr, 2012).

To determine whether either of these possibilities explain the short-term plasticity time course at L4-L2/3 synapses requires information about responses from single synapses. However, each L4 neuron makes ∼5 synaptic contacts onto individual L2/3 neurons (Feldmeyer et al., 2002; Silver et al., 2003), and extracellular stimulation likely recruits multiple presynaptic fibers, making resolution of single-synapse responses impossible through electrophysiological recordings alone. Therefore, we turned to functional Ca^2+^ imaging to identify and monitor synaptic responses at individual L2/3 synapses (Emptage et al., 1999; Yuste et al., 1999; Oertner et al., 2002; Chiu and Carter, 2022).

L2/3 neurons were patched with internal solution containing Alexa Fluor 594 (10 µM) and the Ca^2+^-sensitive fluorescent dye Fluo-5F (300 µM) to image neuronal morphology and Ca^2+^ signals, respectively (Fig. 5*A*). After ≥30 min in the whole-cell configuration to allow for equilibration of the fluorescent dyes, we searched the dendritic arbor for local, transient increases in the Ca^2+^-sensitive fluorescence signal in response to L4 stimulation (Fig. 5*B*). These Ca^2+^ transients arise primarily from NMDAR activity subsequent to presynaptic release of a glutamate-filled vesicle (Chiu and Carter, 2022; Landau et al., 2022) and could be readily detected, as shown in the example in Figure 5. Ca^2+^ transients were restricted to small neuronal compartments; in experiments where concurrent measurements in the neighboring dendritic shaft were possible, these showed smaller, delayed fluorescence increases consistent with Ca^2+^-bound dye diffusing from the synaptic compartment (Sabatini et al., 2002; Chiu and Carter, 2022; Fig. 5*B*). This optical detection method could distinguish between successes and failures on a trial-to-trial basis (Fig. 5*B*), allowing estimation of the probability of release for single synaptic contact sites (Emptage et al., 1999; Oertner et al., 2002; Enoki et al., 2009; Jensen et al., 2021):

$$P_{r}=\frac{\mathrm{num}\;\mathrm{successes}}{\mathrm{num}\;\mathrm{trials}}.$$

On average, the mean geometric distance of the measured synapses was 66.9 ± 5.3 µm from the soma, similar to that measured from anatomical reconstructions from synaptically coupled L4-L2/3 pairs (Feldmeyer et al., 2002). To examine the short-term plasticity of synaptic release, we recorded responses to pairs of stimuli that were delivered at ISI_50_ and ISI_500_ (Fig. 5*B*). Responses were sorted into four possible outcomes: two release failures (*R*_00_), a failure followed by a success (*R*_01_), a success followed by a failure (*R*_10_), or two successes (*R*_11_). In each experiment, both ISIs were tested, and blocks of trials were interleaved.

To obtain a single estimate of the initial *P_r_* for each synapse, we included all trials irrespective of ISI, or:

$$P_{\mathrm{init}}=\frac{\left(R_{10}+R_{11}\right)}{N_{\mathrm{total}}}.$$

For the second stimulation, *P_r_* was calculated as follows:

$$P_{\mathrm{ISI}}=\frac{\left(R_{01}+R_{11}\right)}{N_{\mathrm{ISI}}},$$

where *N*_ISI_ is the total number of trials for a given ISI. The average *P*_init_ was 0.78 ± 0.03 (Fig. 5*D*; *n* = 34), which is in line with previous estimates of L4-L2/3 release probability using synaptic failure rates in paired synaptic recordings (Feldmeyer et al., 2002), multiple-probability fluctuation analysis (Silver et al., 2003), or the MK-801 blocking time course (Castro-Alamancos and Connors, 1997).

To determine whether the likelihood of seeing a response was the same for pairs of stimuli at the two ISIs, we compared *P*_50_ and *P*_500_ with *P*_init_ (Fig. 5*E*; *F*_(2,66)_ = 7.821; *p* = 0.0009; repeated-measures ANOVA; *P*_50_: 0.76 ± 0.03, *p* > 0.05; *P*_500_: 0.69 ± 0.03, *p* < 0.01; *n* = 34 vs *P*_init_ using Dunnett’s test). Normalizing *P*_50_ and *P*_500_ to *P*_init_ (Fig. 5*F*; *P*_50_: 0.99 ± 0.04; *P*_500_: 0.88 ± 0.02; *n* = 34) showed that *P_r_* at the different ISIs followed the same pattern as the PPR of the evoked responses from these trials (comparison with initial EPSC: ISI_50_: 0.99 ± 0.04; ISI_500_: 0.79 ± 0.02; *n* = 34), indicating that an ISI-dependent change in *P_r_* can account for short-term plasticity at this synapse.

To distinguish whether this reflects the contribution of a subset of low-*P_r_*, facilitating synapses (Hessler et al., 1993; Rosenmund et al., 1993; Castro-Alamancos and Connors, 1997), we examined responses measured in individual synapses. To quantify how likely it was that *P_r_* deviated from *P*_init_ for each ISI, we compared *P*_50_ and *P*_500_ to the 95% confidence interval (CI) of *P*_init_ for each recorded synapse using the inverse binomial relationship. The majority of *P*_50_ values were within the 95% CI of *P*_init_ (18/34), and equal numbers of synapses fell above (8/34) and below (8/34) the CI (Fig. 5*F*). In contrast, most measurements of *P*_500_ fell below the 95% CI (20/34), roughly one third were within (12/34) and only two were above, indicating that depression of synaptic responses at ISI_500_ can be attributed to an ISI-dependent decrease in *P_r_*. The finding that most synapses had an unchanged *P_r_* for two APs within 50 ms indicates that the lack of plasticity at this ISI cannot be ascribed only to the summation of separate populations of facilitating and depressing synapses. Moreover, *P*_init_ for this subset of synapses was high (Fig. 5G; 0.81 ± 0.04; *n* = 18), and the ratio of *P*_500_ to *P*_init_ (0.92 ± 0.03) was nearly identical to the average PPR of evoked responses measured in Figure 1 (0.91 ± 0.04; *n* = 19).

The above analyses show that individual L4-L2/3 synapses release a vesicle with high initial probability and can release a second vesicle at short intervals with roughly equivalent probability, yet display a decrease in *P_r_* at longer intervals. Because this can be observed within single synapses, and initial *P_r_* is high at these synapses, there must be distinct molecular determinants of short-term plasticity beyond whether a synapse has an initially high or low *P_r_*.

### The high probability of release at short ISIs requires synaptotagmin 7

Recent studies have found that synaptotagmin 7 (Syt7) functions as the Ca^2+^ sensor for short-term facilitation in many synapses (Jackman et al., 2016; Chen et al., 2017; Weyrer et al., 2021). Although the L4-L2/3 synapse does not show facilitation (Fig. 1), we hypothesized that a facilitation-like process could enhance release at short intervals, counteracting depression. To test this, we assessed L4-L2/3 short-term plasticity in mice lacking Syt7 (Maximov et al., 2008; Syt7-KO) and their wild-type littermates (Syt7-WT), with experimenters blind to the genotype (Fig. 6).

In recordings from Syt7-KO mice, the PPR was significantly lower than in Syt7-WT mice at all ISIs below ISI_1000_ (for ISI_10_, 0.78 ± 0.02 vs 1.33 ± 0.07, *p* < 0.001; ISI_20_, 0.72 ± 0.03 vs 1.24 ± 0.06, *p* < 0.001; ISI_50_, 0.72 ± 0.02 vs 1.11 ± 0.05, *p* < 0.001; ISI_100_, 0.70 ± 0.02 vs 0.97 ± 0.05, *p* < 0.001; ISI_200_, 0.70 ± 0.02 vs 0.85 ± 0.04, *p* < 0.01; ISI_500_, 0.72 ± 0.02 vs 0.87 ± 0.04, *p* = 0.001; *n* = 23 and 24 recordings from Syt7-KO and Syt7-WT mice, respectively; *t* test with Holm–Sidak correction for multiple comparisons). Most strikingly, the shortest ISIs showed pronounced depression (Fig. 6*B*). Subtracting the average PPR measured in Syt7-KO from the Syt7-WT gave a response difference that was well fitted by a single exponential function with a decay time constant of 101.4 ms (Fig. 6*C*), similar to the kinetics of Syt7-dependent paired-pulse facilitation measured in other synapse types (Jackman et al., 2016; Huson and Regehr, 2020; Weyrer et al., 2021).

Given these results, we expected to observe a larger number of release failures at ISI_50_ in Syt7-KO synapses. To test this, we employed the functional Ca^2+^ imaging approach to measure synaptic release probabilities in Syt7-KO and Syt7-WT mice. Initial release probability in Syt7-KO synapses tended to be higher than in Syt7-WT synapses (Fig. 6*D*), but one outlier (*P_r_* = 0.37) was at odds with this observation (Syt7-WT *P_r_* 0.81 ± 0.04; *n* = 11; Syt7-KO *P_r_* 0.84 ± 0.06; *n* = 9; *p* = 0.59; unpaired *t* test). Comparison between genotypes (Fig. 6*E,F*) revealed that the relative change in *P_r_* differed at ISI_50_ but not ISI_500_ (*P*_50_/*P*_init_: 0.95 ± 0.04 vs 0.81 ± 0.06, *p* = 0.04; *P*_500_/*P*_init_: 0.86 ± 0.04 vs 0.81 ± 0.06, *p* = 0.16, *n* = 11 and *n* = 9 for Syt7-WT and Syt7-KO, respectively; unpaired *t* test), consistent with the conclusion that Syt7 shapes short-term plasticity at brief ISIs by limiting synaptic depression.

Syt7 has also been implicated in promoting asynchronous synaptic release (Wen et al., 2010; Bacaj et al., 2013; Luo et al., 2015). Asynchronous release has not been described in L4-L2/3 synapses, but the observation of Syt7-dependent release raises the possibility of this mode of release contributing to synaptic transmission. However, the number of traces that showed asynchronous events in our imaging experiments was very low in both the Syt7-KO (5/804 traces, 0.62%) and Syt7-WT (6/923 traces, 0.65%) synapses, both within the 95% confidence interval of each other.

Taken together, these data indicate that short-term synaptic plasticity at L4-L2/3 synapses arises from the combination of two distinct modes of release: a basal mechanism that has a high (∼0.8) release probability that rapidly depresses and recovers over seconds, and a Syt7-dependent form of release, which contributes to a high probability release for ∼100 ms after the initial stimulation. Both mechanisms coexist at individual synapses, and together shape the short-term plasticity time course.

## Discussion

Synapses between L4 and L2/3 neurons in somatosensory cortex link sensory input from the thalamus to cortical networks (Staiger and Petersen, 2021) and are important for establishing receptive field properties of L2/3 neurons in cortical barrel columns (Feldman and Brecht, 2005). We found that short-term plasticity at L4-L2/3 synapses arises from intrinsic, activity-dependent changes in *P_r_* at single synapses. Moreover, we discovered that Syt7 is required to support high *P_r_* for a second stimulation at very brief ISIs.

### Synaptotagmin 7 counteracts depression

Synaptotagmins are essential for coupling Ca^2+^ influx with vesicle release (Brose et al., 1992; Wolfes and Dean, 2020). Although synchronous vesicle release in the forebrain (Geppert et al., 1994; Fernández-Chacón et al., 2001) is mediated by synaptotagmin 1 (Syt1), Syt7 has the highest apparent Ca^2+^ affinity among the synaptotagmin family (Sugita et al., 2002; Bacaj et al., 2013). Among its proposed functions (MacDougall et al., 2018; Huson and Regehr, 2020), Syt7 is a crucial component in short-term synaptic facilitation (Jackman et al., 2016). Although L4-L2/3 responses do not facilitate, we found that Syt7-mediated release occurs at this synapse.

The most conspicuous effect of this is that L4-L2/3 responses do not depress for pairs of stimuli delivered within ∼100 ms. This is notable because these synapses respond with such high probability to the initial stimulus; at other high-*P_r_* synapses (Dittman and Regehr, 1998; Hashimoto and Kano, 1998; Murphy et al., 2004) and according to the model of short-term synaptic depression due to vesicle depletion (Debanne et al., 1996; Zucker and Regehr, 2002; Foster and Regehr, 2004), PPD is pronounced during this time window. Instead, Syt7-mediated release offsets the underlying short-term depression. Syt7 also plays a key role in asynchronous release (Wen et al., 2010; Bacaj et al., 2013; Luo et al., 2015; Turecek and Regehr, 2018; Huson and Regehr, 2020); however, asynchronous release was not apparent in our experiments. Moreover, asynchronous release may be more prevalent at low-*P_r_* synapses (Atluri and Regehr, 1998; Szapiro and Barbour, 2007; Best and Regehr, 2009; Mendonça et al., 2022), which could account for the lack of asynchronous release here. This shows that Syt7 can be active at high-*P_r_*, depressing synapses in addition to synapses that facilitate or display asynchronous release, and the effect of Syt7 may depend on the unique environment present in different synapses.

One example is high-*P_r_* Purkinje cell (PC) to deep cerebellar nucleus (DCN) synapses (Turecek et al., 2017). There depression is counteracted by Syt7-dependent facilitation that increases *P_r_* of the remaining vesicles to effect frequency-independent response magnitudes. In contrast to PC-DCN synapses, L4-L2/3 synapses do not display PPD at short ISIs in physiological Ca^2+^. Furthermore, L4 neurons are unlikely to have prolonged bouts of high-frequency firing, instead firing one to several APs in response to whisker deflections during natural whisking behavior (Crochet et al., 2011; Hires et al., 2015). Syt7-dependent release at L4-L2/3 synapses creates a narrow time window permissive to repetitive, high-*P_r_* transmission, “delaying” the manifestation of short-term depression.

While short-term plasticity at L4-L2/3 synapses initially appeared inconsistent with the depletion model of depression, our results from Syt7-KO mice revealed a basal, high-probability, Syt7-independent mode of release, which was consistent with the depletion model of short-term depression (Debanne et al., 1996; Dobrunz and Stevens, 1997; Zucker and Regehr, 2002).

### Facilitation and depression coexist within single synapses

On average, an L4 neuron makes ∼5 synapses onto a given L2/3 neuron (Feldmeyer et al., 2002; Silver et al., 2003); thus, evoked responses represent many activated synapses. The combination of facilitation and depression seen at these synapses could occur through temporal summation of spatially distinct synapses; however, by imaging we found that both depression and facilitation combine in the same synapse. It remains to be discovered how this combination of seemingly independent release mechanisms, which may contribute to the greater variability in PPR at the shortest intervals, is regulated. In contrast to Syt1, which is primarily found on synaptic vesicles (Matthew et al., 1981; Brose et al., 1992), in neurons Syt7 primarily associates with the plasma membrane (Sugita et al., 2001; Takamori et al., 2006; Vevea et al., 2021). Syt7 deletion can decrease the number of docked vesicles (Tawfik et al., 2021; Wu et al., 2023), indicating that it may increase priming or decrease depriming rates (Tawfik et al., 2021). However, the factors determining whether Syt7 interacts with a given synaptic vesicle are unknown (Huson and Regehr, 2020).

The contribution of Syt7 in high-probability synapses underscores the fact that initial *P_r_* is not the only determinant shaping short-term synaptic plasticity. By using two distinct release mechanisms, the probability of the initial release event can be independent of the subsequent, Syt7-dependent release event. Release is more likely after Syt7 is activated by an initial Ca^2+^ increase, which in synapses with low initial *P_r_* manifests as facilitation, while in those with high initial *P_r_* counteracts depression. In both cases, the synapse preferentially conveys brief, high-frequency input. Syt7 is not simply a mediator of facilitation, but rather a high-affinity sensor that requires more than a brief Ca^2+^ rise to promote vesicle fusion. In this sense, Syt7 functions in its own rite as a coincidence detector.

Comparisons of PPR before and after experimental manipulations are commonly used to infer whether a change in *P_r_* has taken place (Debanne et al., 1996; Dobrunz and Stevens, 1997; Zucker and Regehr, 2002). However, measuring a change in PPR does not provide direct evidence about whether the first or the second response (or both) has changed and is therefore not necessarily a reliable indicator of *P_r_*. For example, manipulations that lower *P_r_* can increase PPR, even turning depressing synapses into facilitating ones (Murphy et al., 2004; Weyrer et al., 2021). But this is not always true: in prefrontal cortex, for example, dopamine can affect *P_r_* without changing PPR (Burke et al., 2018). Our finding that mechanisms for both types of short-term plasticity coexist in single synapses adds further complexity to the interpretation of PPR changes, as a given manipulation could affect the two distinct modes of release differently. For example, we found that neuromodulators did not account for the short-term depression seen at ISIs between 100 and 1,000 ms (Fig. 2). However, blocking mGluRs and GABA_B_Rs increased PPR at short intervals. Having two mechanisms of release may thus endow a synapse with greater flexibility to fine-tune release through modulatory signals or long-term plasticity.

### Functional implications

By effectively increasing or decreasing the weight of an AP based on prior activity, short-term plasticity filters information at a synapse (Abbott and Regehr, 2004; Regehr, 2012), with facilitation acting as a high-pass filter, and depression as a low-pass filter (Tsodyks and Markram, 1997). Both their high *P_r_*, which allows reliable information transfer with a single stimulus, and the short-term depression observed at longer intervals confer low-pass filtering properties to L4-L2/3 synapses, which is important for sensory adaptation (Latimer et al., 2019; Katz and Lampl, 2021). Syt7-mediated release adds a high-pass filter, creating a notch filter-like effect on incoming signals. This has implications not only for the integration and propagation of the postsynaptic potential, but, as these processes occur in the same synapse, for postsynaptic molecular signaling pathways as well.

Most L4-L2/3 synapses reside on spines (Feldmeyer et al., 2002), which serve as biochemical and electrical signaling units (Müller and Connor, 1991; Yuste and Denk, 1995; Yasuda, 2017). These synapses are particularly favorable to postsynaptic NMDAR signaling, as NMDARs in L2/3 pyramidal neurons are activated by a single stimulus (Chiu and Carter, 2022) and conduct Ca^2+^ even at resting membrane potentials (Fig. 5). NMDARs activate myriad intracellular signaling pathways in spines (Yasuda, 2017), are required for long-term plasticity induction at L4-L2/3 synapses (Fox, 2002; Bender et al., 2006b; Glazewski et al., 2017), and can even signal in an ion-independent manner (Carter and Jahr, 2016). Ca^2+^ influx can be instructive for the direction of plasticity (Nevian and Sakmann, 2006) and can influence spine morphology (Oertner and Matus, 2005), which in turn can affect signaling properties (Hayashi and Majewska, 2005; Yasuda, 2017). Given their initial high *P_r_* and Syt7 mode of release, L2/3 spines receiving L4 inputs are likely to be exposed to multiple and overlapping signaling cascades, which could be uniquely important for L2/3 homeostasis and plasticity.

The function of short-term plasticity is often considered in terms of its net effect on the whole-cell response. It is, after all, the sum total of all the synaptic inputs that determine whether the postsynaptic cell fires an AP, propagating the incoming signal through the network. Our findings highlight one way in which short-term plasticity can affect not only the membrane potential of the postsynaptic neuron, but also local biochemical signaling at the level of a single spine. Whether the particular configuration that allows L4-L2/3 synapses to transmit successive activity with high fidelity at very low and very high frequencies is found at other synapses is an open question. The previously unrecognized combination of vesicle release properties found here should stimulate new questions and hypotheses with respect to activity-dependent plasticity and synapse-specific information processing.
